# Adolescent Mothers in Eastern and Southern Africa: An Overlooked and Uniquely Vulnerable Subpopulation in the Fight Against HIV

**DOI:** 10.1016/j.jadohealth.2021.12.012

**Published:** 2022-06

**Authors:** Allison K. Groves, Luwam T. Gebrekristos, Patrick D. Smith, Kirsten Stoebenau, Marie C. Stoner, Wole Ameyan, Alex C. Ezeh

**Affiliations:** a Dornsife School of Public Health, Drexel University, Philadelphia, Pennsylvania; b Department of Behavioral and Community Health, School of Public Health, University of Maryland, College Park, Maryland; c Women's Global Health Imperative, RTI International, Berkeley, California; d Global HIV, Hepatitis and Sexually Transmitted Infections Programmes, World Health Organization, Geneva, Switzerland

**Keywords:** Adolescent pregnancy, Adolescent motherhood, HIV prevention, Africa, Adolescent girls and young women

## Abstract

**Purpose:**

Adolescent girls (10–19 years) in Eastern and Southern Africa face a high risk of pregnancy and HIV infection. However, few studies have examined whether the profound developmental, social, and economic changes that accompany adolescent motherhood contribute to HIV risk. This study examines the intersection between adolescent motherhood and HIV infection across 10 Eastern and Southern African countries, where over half of all HIV infections occur among adolescent girls.

**Methods:**

To evaluate whether adolescent motherhood is associated with HIV infection, we used Demographic and Health Survey data on girls (15–19 years) with HIV test results (N = 19,932) from Eswatini, Kenya, Lesotho, Malawi, Mozambique, South Africa, Tanzania, Uganda, Zambia, and Zimbabwe. We examined unweighted bivariate and multivariable associations between adolescent motherhood and HIV using mixed effects logistic regression models that included a country-level random intercept. We examined heterogeneity in the association by testing country-level random slopes using a likelihood ratio test and used intraclass correlation to measure the proportion of total variance explained at the country level.

**Results:**

Nearly one fifth of adolescent girls were mothers (range: 9.80%–38.90%), and the HIV prevalence among all adolescent girls was 3.3% (range: 1.03%–10.07%). Relative to nonmothers, adolescent mothers were, on average, older, poorer, and more likely to be married, rural dwellers, and household heads. Adolescent motherhood was positively associated with HIV infection in bivariate and multivariable analyses (odds ratio: 1.87; 95% confidence interval: 1.57–2.23; adjusted odds ratio: 1.53; 95% CI: 1.24–1.89).

**Discussion:**

Among adolescents with HIV test results, we observed a robust association between adolescent motherhood and HIV infection across 10 high-burden countries.

**Implications and Contribution Summary Statement**

Although structural factors may increase adolescent girls' risk of motherhood and HIV, few studies have explored the association between motherhood and HIV. This analysis, alongside growing evidence of poor outcomes for adolescent mothers across HIV prevention and care cascades, highlights a need for tailored HIV prevention interventions for adolescent mothers.

Eastern and Southern Africa have some of the highest rates of adolescent pregnancy worldwide [1]. Specifically, more than one in 5 adolescents on the African continent have given birth before the age of 20 years [2], and many of these pregnancies are unplanned [3,4]. In addition, despite extensive work to prevent unintended pregnancy among adolescents, adolescent pregnancy rates are projected to increase over the next decade [5].

Concurrently, adolescent girls (defined by the World Health Organization as those between 10 and 19 years) in Eastern and Southern Africa are among those at the highest risk of HIV globally [6]. Eastern and Southern Africa accounts for 43% of all new HIV infections despite accounting for less than 10% of the world's population [7]. Within this region, 15% of all new infections occur among adolescents aged 10–19 years, with 83% of adolescent infections occurring in girls [7]. Further underscoring the HIV risk faced by adolescent girls in this region, a recent meta-analysis found that HIV incidence among female adolescents exceeded that of males across all settings [8]. Moreover, over half of all HIV infections among adolescent girls globally occur in just 10 countries in Eastern and Southern Africa: Eswatini, Kenya, Lesotho, Malawi, Mozambique, South Africa, Tanzania, Uganda, Zambia, and Zimbabwe [7,9].

Structural drivers of HIV infection, including exposure to poverty, gender inequality, and violence, may increase the susceptibility of adolescent girls in Eastern and Southern Africa to both pregnancy and HIV infection [10,11]. Simultaneously, although education and adolescent-friendly health services are recommended as key protective factors, gaps in access to and quality of adolescent-friendly health services have hampered efforts to reduce persistent inequities [12]. Although the influence of such structural drivers for both HIV and pregnancy has been well-examined among adolescent girls, few studies have considered whether and how changes in life circumstances that accompany adolescent motherhood influence HIV risk.

Theoretically, adolescent motherhood may impact HIV risk through numerous pathways. First, biological changes in pregnancy, including hormonal changes which might affect immune responses or the genital tract mucosa, may increase HIV acquisition [13]. Second, the onset of adolescent motherhood may contribute to heightened economic and social vulnerability. For adolescents who are unmarried and living in their natal households, costs associated with adolescent pregnancy may cause economic strain [14], which may in turn impact adolescent mothers' sexual partnerships. In contexts where premarital pregnancy is stigmatized, some adolescent girls may be ‘chased’ from their natal homes, leading adolescents to raise children on their own or to establish informal partnerships with fathers of their children [15]. Young mothers who have had a premarital birth remain single longer than young nulliparous, never-married women [16,17], and limited evidence suggests the unions they do form are less formal [16,18], which could result in increased exposure to sexual partners and, therefore, HIV. That is, unmarried adolescent mothers may prioritize the formation and/or maintenance of sexual partnerships that offer the promise of economic gain or stability. Economic and/or material imbalances within such relationships may affect adolescent mothers' negotiating power, decreasing their ability to negotiate safe sex and/or increasing their risk of intimate partner violence [18]. Simultaneously, married adolescents may face slightly different constraints in managing HIV risk. In particular, they may have limited ability to negotiate the conditions around sex and, in some cases, their partner’s infidelity [19]. Third, one negative consequence of adolescent motherhood is school dropout, which itself is associated with increased HIV acquisition among young women in HIV-endemic settings [20]. Finally, pregnant teenagers who are marked by health care workers, family members, and/or the broader community as ‘bad girls’ because of their adolescent pregnancy may subsequently face stigma and discrimination [21]. Such stigma may negatively impact their mental health, engagement in care, and ability to access necessary HIV prevention tools, like pre-exposure prophylaxis (PrEP), both during and after pregnancy.

Despite a compelling rationale for why adolescent mothers may face increased HIV risk relative to nonmothers, the relationship remains surprisingly underexamined. To date, two longitudinal studies (one in Uganda and one in South Africa) have shown positive associations between adolescent motherhood and incident HIV infection [22,23]. In contrast, another longitudinal study that examined whether adolescent motherhood predicted incident HIV among school-going adolescent girls in South Africa did not find evidence of an effect [24]. However, several methodological challenges with extant studies constrain interpretation and generalizability of the findings. Specifically, all three studies used convenience samples; measurement of the exposure (adolescent motherhood) differed across studies, and one study did not adjust for potential confounders, which may have produced a spurious association between the variables. Many other studies examining incident HIV infection among adolescent girls and young women (AGYW) more broadly include pregnancy as a covariate but not as the primary focus of their analyses (as one example, see Kilburn et al., 2018) [25].

Therefore, this study's purpose is to test the association between adolescent motherhood and HIV across 10 countries in Eastern and Southern Africa. Our approach achieves two aims: it broadens the geographic generalizability of earlier findings across varying epidemiological, political, and sociocultural contexts, and it examines differences in the association across different ages of adolescents. Understanding whether adolescent motherhood is associated with HIV in HIV-endemic settings is important not only for ensuring the well-being of adolescent mothers and their children, but also for achieving the Sustainable Development Goals and the Global Strategy for Women's, Children's and Adolescents' Health by 2030.

## Methods

### Data

Cross-sectional, nationally representative data were drawn from the Demographic and Health Surveys (DHSs) and AIDS Indicator Surveys (AISs) (both conducted by The DHS Program using comparable designs) of the following 10 countries in Eastern and Southern Africa, which together account for more than half of all HIV infections among AGYW globally: Kenya (2008–2009), Lesotho (2014), Malawi (2015–2016), Mozambique (2015), Eswatini (2006–2007), Tanzania (2015–2016), Uganda (2011), Zambia (2018), Zimbabwe (2015), and South Africa (2016) [26]. Only the most recent survey containing HIV testing results for each country was used for this analysis. Therefore, the analytic data set includes data collected from 2006 to 2016.

The DHS collects data on health and sociodemographic characteristics using a two-stage cluster sampling design. Clusters are randomly selected within strata, which are determined by geographic location and residence type. Within the selected clusters, households are randomly selected. Data are collected from interviews with all individual household members aged 15–49 years. For this analysis, only interviews with adolescent girls aged 15–19 years were used, as adolescents under 15 years are not included in standard DHS samples. In addition to interviews, a subsample of respondents in each country completes HIV testing, which is voluntary and subject to a separate consent process. Blood spots are collected for household members participating in HIV tests, and positivity is determined by enzyme-linked immunosorbent assay (ELISA). All positive tests are retested, and 5–10 percent of negative tests are retested with a second ELISA. For those with discordant results, a new ELISA or Western blot is performed. Further details on study procedures, sampling, questionnaires, and HIV testing quality assurance can be found elsewhere [26]. Study procedures and questionnaires for all DHSs and AISs are approved by the ICF Institutional Review Board and individual institutional review boards in the host countries. The present study was reviewed by the human research protection program at Drexel University. Because the protocols for conducting DHSs ensure the anonymity of individual-level data, this study was considered secondary research and was exempted from full human subjects' review.

### Measures

Adolescent motherhood was measured using a single item asking participants the number of children they have given birth to. Individuals who reported having given birth to at least one child were coded as adolescent mothers. Individuals who reported having not given birth were coded as nonmothers.

The outcome variables include HIV status (HIV-positive vs. HIV-negative) and country-level HIV prevalence.

The sociodemographic covariates of interest include age (continuous and categorical), current marital status (married vs. unmarried), female head of the household (yes vs. no), relationship to the head of the household (coded as self, wife, family/relative, other), type of residence (rural vs. urban), and socioeconomic status, which was measured through a wealth index (i.e., a score of wealth calculated using principal components analysis on the household's ownership of selected assets). Scores were divided into wealth quintiles, with one being the lowest and five being the highest.

### Analysis

Our analytic sample was restricted to adolescent girls between the ages of 15–19 years with conclusive HIV results (N = 19,932). We first described sociodemographic characteristics of adolescent girls across the 10 countries. Next, we examined adolescent motherhood and HIV weighted prevalence across countries using DHS weights for each country. To examine differences in HIV prevalence by adolescent motherhood and age, we measured age categorically. Third, we examined unweighted bivariate and multivariable associations between adolescent motherhood and HIV using mixed effects logistic regression models with a country-level random intercept to account for country-level clustering [27]. Multivariable mixed effects models included fixed effects for age (categorical), marital status, type of residence, relationship to the head of the household, and wealth quintiles because they were considered a priori confounders of adolescent motherhood and HIV. We calculated the variance inflation factor to ensure there was no multicollinearity among independent variables (variance inflation factor <5) [28]. We measured the proportion of total variance that was explained at the country level using intraclass correlation [29]. Furthermore, we tested country-level random slopes using a likelihood ratio test to examine whether the association between adolescent motherhood and HIV infection varied by country. All analyses were conducted using SAS 9.4 [30] and RStudio [31,32].

## Results

### Sample description

Among all adolescent girls included in the analysis, nearly one in five were mothers (17.4%). Moreover, among mothers, over 15% had given birth to more than one child. The mean age of participants was 17.0 years (standard deviation 1.4) (Table 1). More adolescent girls resided in rural areas than in urban areas (71.4%). Approximately one in 10 adolescent girls were currently married (12.1%).

**Table 1:** Sociodemographic characteristics of 15- to 19-year-old adolescent mothers and nonmothers across 10 countries

Variables	All adolescent girls (N = 19,932)	Nonmothers (N = 16,472)	Mothers (N = 3,460)	*p* value
	Mean (sd), % (n)			
Outcome				
HIV prevalence	3.3 (658)	2.8 (459)	5.8 (199)	<.0001
Sociodemographic characteristics				
Age	17.0 (1.4)	16.7 (1.4)	18.0 (1.1)	<.0001
Currently married	12.1 (2,410)	5.2 (850)	45.1 (1,560)	<.0001
Female head of the household	35.3 (7,041)	35.8 (5,900)	33.0 (1,141)	.002
Relationship to the head of the household				<.0001
Self	2.8 (561)	2.0 (335)	6.5 (226)	
Wife	7.5 (1,492)	2.9 (479)	29.3 (1,013)	
Family relative	86.5 (17,237)	91.5 (15,078)	62.4 (2,159)	
Other	3.2 (642)	3.5 (580)	1.8 (62)	
Wealth Quintiles				<.0001
Lowest	16.3 (3,257)	14.7 (2,419)	24.2 (838)	
Second	17.7 (3,533)	16.7 (2,747)	22.7 (786)	
Middle	19.9 (3,963)	19.5 (3,210)	21.8 (753)	
Fourth	22.1 (4,412)	22.7 (3,744)	19.3 (668)	
Highest	23.9 (4,767)	26.4 (4,352)	12.0 (415)	
Rural residence	71.4 (14,223)	70.0 (11,534)	77.7 (2,689)	<.0001

sd = standard deviation.

Adolescent mothers were, on average, significantly older than nonmothers (18.0 years vs. 17.0 years). Adolescent mothers were also more likely to be married than nonmothers (45.1% vs. 5.2%) and more likely to be in lower wealth quintiles than their nonmother peers.

### The association between adolescent motherhood and HIV infection

**Figure 1:**
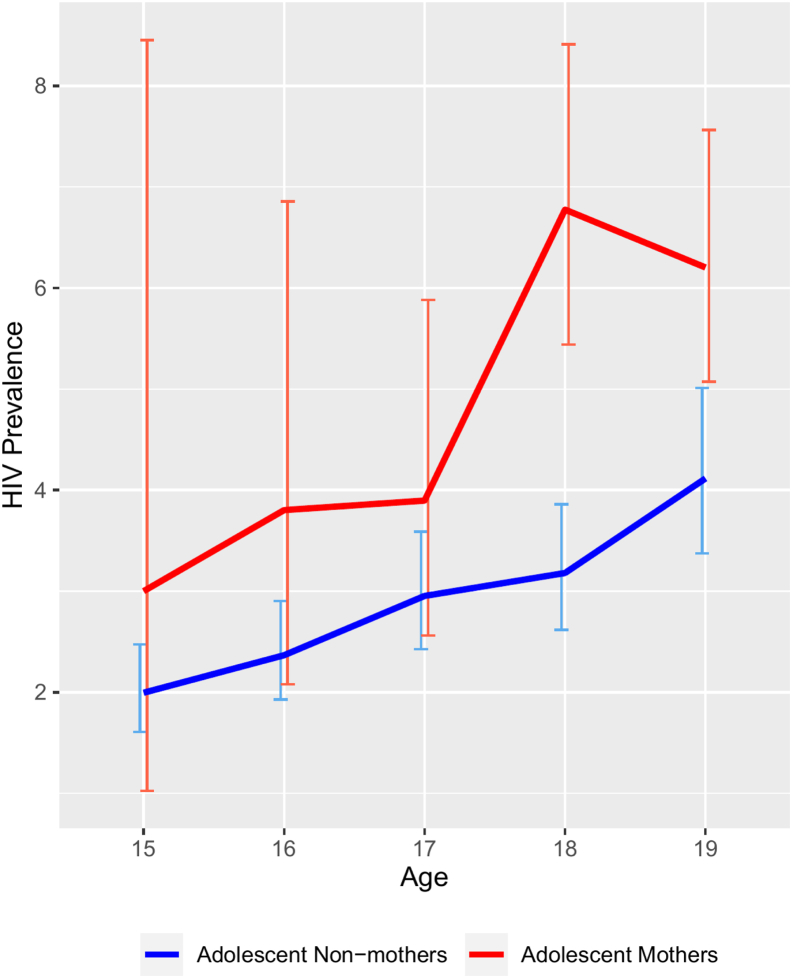
HIV prevalence of adolescent mothers and nonmothers by age.

Across all 10 countries, the HIV prevalence was 3.3% (Table 1). The raw distribution shows a higher HIV prevalence among adolescent mothers (5.8%; 95% confidence interval [CI]: 5.00–6.58) than among nonmothers (2.8%; 95% CI: 2.54–3.05). Relatedly, Figure 1 shows the HIV prevalence for adolescent mothers at each age. Although HIV prevalence estimates for adolescent mothers were higher than HIV prevalence estimates for nonmothers across all ages, the difference between these groups was only significant among those who were 18 and 19 years old (Figure 1).

As seen in Table 2, which presents the weighted prevalence of HIV and adolescent motherhood for each country, the prevalence of adolescent motherhood varied considerably across countries. Specifically, the prevalence of adolescent motherhood ranged from 9.80% (Zimbabwe) to 38.90% (Mozambique). HIV prevalence among adolescent girls also varied (ranging from 1.0% [Tanzania] to 10.1% [Eswatini]), which is unsurprising given the national-level variation in HIV prevalence across countries. Adolescent marriage also varied across country contexts, with the lowest rates of marriage occurring in South Africa (2.33%) and the highest occurring in Mozambique (25.30%) (Table 2).

**Table 2:** Weighted country-level prevalence for adolescent motherhood, HIV, and adolescent marriage

Country	Unweighted N	Adolescent motherhood (%)	HIV (%)	Currently married (%)
Eswatini	1,195	18.59	10.07	3.54
Kenya	798	14.15	2.74	9.01
Lesotho	791	14.99	5.44	16.92
Malawi	1,657	23.34	3.39	22.88
Mozambique	1,369	38.90	6.54	25.30
South Africa	471	15.72	6.02	2.33
Tanzania	4,234	10.39	1.03	10.03
Uganda	4,453	12.45	2.37	8.01
Zambia	2,986	24.37	2.58	14.14
Zimbabwe	1,978	9.80	3.95	16.54

Table 3 presents the bivariate and multivariable associations between adolescent motherhood and HIV serostatus. In bivariate mixed-effects analysis (model 1), adolescent motherhood was positively associated with HIV infection (odds ratio [OR]: 1.87; 95% CI: 1.57–2.23). In multivariable mixed-effects analysis (model 2), the association was attenuated, but still significant (adjusted OR [AOR]: 1.53; 95% CI: 1.24–1.89). In multivariable analysis, we found no association between marital status and HIV infection (AOR: 0.96; 95% CI: 0.72–1.27) or between wealth strata and HIV infection, controlling for all other covariates and motherhood. However, as seen in model 2, compared with adolescents who were heads of their households, those living with a spouse and those living with a family member or relative were marginally (AOR: 0.68; 95% CI: 0.44–1.05) and significantly (AOR: 0.62; 95% CI: 0.44–0.88) less likely to be HIV-positive. Rural residence was negatively associated with HIV infection (AOR: 0.70; 95% CI: 0.44–0.87). Finally, the odds of HIV infection increased with age; specifically, compared with 15-year-old adolescents, 17-year-old adolescents had a 40% increase in odds (95% CI: 1.05–1.87), 18-year-old adolescents had a 78% increase in odds (95% CI: 1.35–2.34), and 19-year-old adolescents had a 96% increase in odds (95% CI: 1.48–2.60) (Table 3).

**Table 3:** Mixed-effects logistic regression models examining the pooled association between adolescent motherhood and HIV (N = 19,932)

Independent variables	Model 1		Model 2^a^	
	OR (95% CI)	*p* value	AOR (95% CI)	*p* value
Adolescent motherhood				
No	Ref		Ref	
Yes	1.87 (1.57, 2.23)	<.0001	1.53 (1.24, 1.89)	<.0001
Age				
15			Ref	
16			1.15 (0.86, 1.55)	.35
17			1.40 (1.05, 1.87)	.02
18			1.78 (1.35, 2.34)	<.0001
19			1.96 (1.48, 2.60)	<.0001
Wealth index				
Poorest			Ref	
Poorer			.86 (0.65, 1.14)	.29
Middle			1.00 (0.76, 1.31)	.71
Richer			0.94 (0.68, 1.28)	.99
Richest			0.94 (0.68, 1.28)	.68
Marital status				
Not married			Ref	
Married			0.96 (0.72, 1.27)	.78
Type of residence				
Urban			Ref	
Rural			0.70 (0.56, 0.87)	.001
Relationship to the head of the household				
Self			Ref	
Wife			0.68 (0.44, 1.05)	.08
Family relative			0.62 (0.44, 0.88)	.008
Other			0.66 (0.38, 1.12)	.12
Random effects	Variance (95% CI)		Variance (95% CI)	
Intercept (country)	0.44 (0.20, 1.27)		0.43 (0.19, 1.23)	

AOR = adjusted odds ratio; CI = confidence interval; OR = odds ratio.

a Controlled for age, wealth index, marital status, type of residence, and relationship to the head of the household.

**Figure 2:**
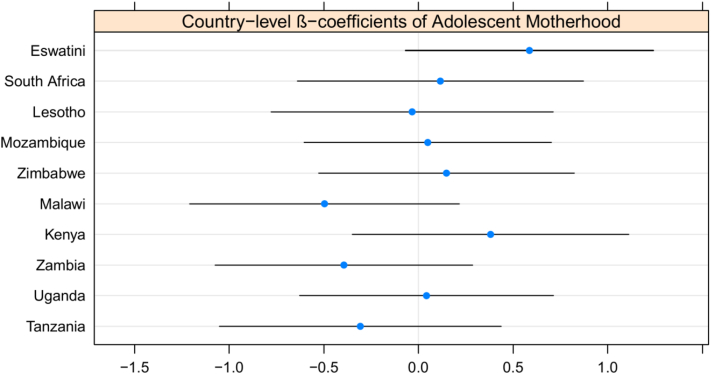
Examining heterogeneity across countries in the association between adolescent motherhood and HIV prevalence.

Finally, we examined heterogeneity in the association between adolescent motherhood and HIV prevalence across country contexts. Specifically, Figure 2 presents the country-level β-coefficient of adolescent motherhood. All country-level CIs cross zero, which suggests that there may not be meaningful heterogeneity across countries in the association (Figure 2). Furthermore, all country-level CIs overlap, which indicates that a random slope is not necessary to account for country variability. The intraclass correlation is 0.035 for adolescent mothers and 0.025 for nonmothering adolescents, suggesting that 3.5% and 2.5% of the total variance is explained at the country level for adolescent mothers and nonmothers, respectively. Finally, results from the likelihood ratio test were insignificant, χ^2^(2) = 6.20, *p* value = .10, meaning the simpler multivariable model (country-level random intercept and no random slope) was the best fitting model [33].

## Discussion

Attention to the HIV risk of adolescents in sub-Saharan Africa has increased substantially over the past decade. However, although mothers comprise a significant subset of adolescents (i.e., in our sample, nearly one in five were adolescent mothers), intersections between adolescent motherhood and HIV have historically been underexamined. We found a robust association between adolescent motherhood and HIV infection among a sample of adolescent girls with HIV test results from 10 African countries. We also found that HIV prevalence was significantly higher among 18- and 19-year-old adolescent mothers than that among nonmothers of the same age. Furthermore, the observed pattern was consistent across countries, despite country-level variation in HIV prevalence. We elaborate on the implications in the following.

First, our findings build on the small body of existing evidence that supports an association between adolescent motherhood and HIV [22,23]. Moreover, our finding that adolescent motherhood is associated with HIV after controlling for marriage, coupled with the fact that marriage itself was not associated with HIV, differs from earlier literature in which marriage was associated with HIV in general and for young women in particular [19,34]. Although adolescent mothers in this analysis were more likely to be married than nonmothers, not all adolescent pregnancies or childbirths occur within the context of marriage [35]. Our focus on adolescent motherhood as the primary exposure was intentional as we theorize that adolescent motherhood impacts HIV risk for both married and unmarried adolescent mothers, albeit through somewhat different pathways. Future longitudinal research to assess temporality and to explicate these pathways is warranted given that intervention implications likely differ for these subgroups.

Despite the growing attention to adolescent mothers' HIV risk, there are notable gaps in evidence-based HIV prevention interventions and service delivery approaches tailored for adolescent mothers [36,37]. Most HIV prevention programs for adolescent girls target factors that precede motherhood (i.e., delayed sexual debut, delayed first pregnancy). Such programs may overlook the HIV-prevention needs of AGYW who are pregnant or intend to become pregnant, despite the likelihood that pregnancy intention is associated with decreased condom use and, thus, HIV risk [38,39]. Furthermore, although combination HIV prevention interventions like DREAMS (Determined, Resilient, Empowered, Mentored, and Safe) may include adolescent mothers as beneficiaries, to our knowledge, no evaluations have examined whether DREAMS has differential reach, acceptability, or outcomes for adolescent mothers. Community-based interventions that specifically target adolescent mothers' distinct social and economic needs (e.g., interventions with content relevant for young married mothers or out-of-school adolescent mothers) are rarer, and few have been rigorously evaluated to date [37].

Relatedly, although numerous interventions have been proposed and tested to strengthen prevention of mother-to-child transmission (PMTCT) care engagement for all mothers-to-be, service delivery gaps persist for the youngest women attending antenatal care [40]. HIV-uninfected adolescent girls are less likely to receive HIV testing [41] and face a higher risk of acquiring HIV during the perinatal period than older women [42]. Furthermore, although PrEP is available during pregnancy in some countries (i.e., South Africa and Kenya), evidence suggests that adolescent girls face greater challenges adhering to PrEP during this time than older women [43], possibly due to barriers imposed by their social context and developmental stage, which may include limited transportation access, restrictive school schedules, and/or difficulty reconciling the deferred and often unseen benefits of PrEP with the potential side effects and stigma associated with PrEP usage [44]. As PrEP is scaled up globally, implementers should ensure that services are accessible and sensitive to the specific needs of adolescent girls in each local context [45,46].

Finally, there is a continued need for interventions and service delivery approaches for pregnant and parenting adolescents living with HIV [40]. Compared with older HIV-infected women, HIV-infected adolescent girls have lower uptake of PMTCT services [41,47], are more likely to transmit HIV to their infants [47], and are more likely to disengage from postpartum HIV care [48]. Moreover, HIV-infected adolescent girls have lower antiretroviral adherence and lower rates of viral suppression than adult women during pregnancy and in the first 12 months postpartum [48]. Although some peer-based models integrated into service delivery (e.g., mentor mothers, mothers-to-mothers, etc.) have documented improvements in health care utilization and health outcomes for adolescents [49], further efforts are needed to scale up adolescent-friendly services and to conduct rigorous implementation science research that describes interventions' impact across settings [50].

There are several study limitations. First, the cross-sectional nature of the data set limits our findings. Consequently, we should not infer causality. Findings are also limited by our inability to include very young adolescent mothers in our sample (given the absence of pregnancy data for adolescents aged between 10 years and 14 years), even though other research shows elevated HIV risk among this subgroup [23]. Findings may also be restricted to those adolescents with HIV test results. Although the AIS for each country uses an established two-stage cluster sample survey to obtain HIV prevalence estimates that are representative at the national and urban/rural level, we acknowledge that there may be differences between adolescent girls who uptake testing (who are included in this analysis) and those who do not. Within the AIS sample, three countries, Zambia, Zimbabwe, and South Africa, had differences in sociodemographic characteristics by HIV testing uptake. However, these differences did not hold among AGYW participants in Zambia or Zimbabwe. Given that there were no significant sociodemographic differences among AGYW who tested compared with those AGYW who did not in nine of the 10 included countries among a data set that is nationally representative, we do not believe our findings are restricted to those adolescents with HIV test results.

Nonetheless, the data highlight that adolescent mothers—who comprise a large and developmentally distinct subset of adolescent girls across settings—are disproportionately affected by HIV among a sample of adolescents who have been tested for HIV. Given relatively low rates of consistent condom usage among adolescent girls [51], pregnancy and HIV infection may theoretically occur simultaneously (or nearly so). Furthermore, HIV infection may precede motherhood, as individuals infected with HIV perinatally may experience significant HIV-related vulnerabilities during adolescence (e.g., orphanhood, stigma) that may, in turn, increase exposure to unsafe sexual partnerships and subsequent early motherhood. Future longitudinal studies are needed to establish temporality and examine pathways through which adolescent motherhood impacts HIV risk.

An additional limitation is that we may have omitted potentially important covariates from our analysis. For example, over half of the sample was missing data on orphanhood. Thus, we did not adjust for orphanhood, although it is a possible confounder. Moreover, given the well-documented challenges with accurate reporting of sexual behavior (e.g., incident HIV infections among young women in a longitudinal cohort study who reported never having had sex, virgin births, etc.) [25], we did not include ‘ever had sex’ as a covariate; however, having had sex may confound the association. Nevertheless, given the programmatic challenges of identifying which teenagers are sexually active, coupled with the fact that teen pregnancy makes sexual activity ‘visible,’ the findings are still actionable. In future research, refinement of measures to identify who is sexually active will facilitate closer examination of the unique effects of adolescent motherhood on HIV.

Despite these limitations, our study is the first to examine the association between adolescent motherhood and HIV prevalence using data across 10 countries with a high burden of HIV and adolescent motherhood. Although there are challenges in combining data across settings with significant epidemiological, political, and sociocultural differences, a multicountry approach highlights the importance of understanding the vulnerability of adolescent mothers to HIV across different African countries. Findings also highlight the need for country-specific research to further understand and address the intersection of HIV and adolescent motherhood. Finally, our study underscores that AGYW are not a monolithic group; as such, subsets of adolescents (e.g., mothers, AGYW who are heads of households, etc.) may face different risks and need different services than their peers. Given the heterogeneity of AGYW and the centrality of stakeholder involvement to effective program improvement and advocacy, future efforts must engage adolescent mothers in addressing current gaps in programming and policy.

### Conclusions

A growing focus on the high HIV risk faced by adolescent girls in sub-Saharan Africa has led to important investments in the health and social welfare of this population. However, researchers and program implementers have often failed to adequately describe or account for the unique needs of adolescent mothers when designing, implementing, and evaluating interventions surrounding HIV prevention and management. The association of adolescent motherhood with HIV, coupled with existing research which suggests this group is particularly vulnerable to poor outcomes across the HIV prevention and care cascades, suggests that targeted interventions, developed in collaboration with adolescent mothers during and after pregnancy, have the potential to profoundly impact the health of adolescent mothers and their children.
